# *Aspergillus oryzae*-Fermented Wheat Peptone Enhances the Potential of Proliferation and Hydration of Human Keratinocytes through Activation of p44/42 MAPK

**DOI:** 10.3390/molecules26196074

**Published:** 2021-10-08

**Authors:** Kyung Man Hahm, See-Hyoung Park, Sae Woong Oh, Ji Hye Kim, Hyun Sook Yeom, Hye Ja Lee, Seoyeon Yang, Jae Youl Cho, Jin Oh Park, Jongsung Lee

**Affiliations:** 1 Molecular Dermatology Laboratory, Department of Integrative Biotechnology, College of Biotechnology and Bioengineering, Sungkyunkwan University, Suwon City 16419, Gyunggi Do, Korea; project@daebongls.co.kr (K.M.H.); hanzeeoo@skku.edu (S.W.O.); chorim1004@skku.edu (S.Y.); 2 Natural Products Laboratory, Daebong LS Co., Ltd., Incheon 21697, Korea; jh.kim2@daebongls.co.kr (J.H.K.); hs.yeom@daebongls.co.kr (H.S.Y.); hj4170@daebongls.co.kr (H.J.L.); 3 Department of Bio and Chemical Engineering, Hongik University, Sejong City 30016, Korea; shpark74@hongik.ac.kr; 4 Molecular Immunology Laboratory, Department of Integrative Biotechnology, College of Biotechnology and Bioengineering, Sungkyunkwan University, Suwon City 16419, Gyunggi Do, Korea

**Keywords:** cell proliferation, skin hydration, *Aspergillus oryzae*, wheat peptone, MAPK

## Abstract

Identifying materials contributing to skin hydration, essential for normal skin homeostasis, has recently gained increased research interest. In this study, we investigated the potential benefits and mechanisms of action of *Aspergillus oryzae*-fermented wheat peptone (AFWP) on the proliferation and hydration of human skin keratinocytes, through in vitro experiments using HaCaT cell lines. The findings revealed that compared to unfermented wheat peptone, AFWP exhibited an improved amino acid composition, significantly (*p* < 0.05) higher DPPH scavenging capability and cell proliferation activity, and reduced lipopolysaccharide-induced NO production in RAW 264.7 cells. Furthermore, we separated AFWP into eleven fractions, each ≤2 kDa; of these, fraction 4 (AFW4) demonstrated the highest efficacy in the cell proliferation assay and was found to be the key component responsible for the cell proliferation potential and antioxidant properties of AFWP. Additionally, AFW4 increased the expression of genes encoding natural moisturizing factors, including filaggrin, transglutaminase-1, and hyaluronic acid synthase 1–3. Furthermore, AFW4 activated p44/42 MAPK, but not JNK and p38 MAPK, whereas PD98059, a p44/42 MAPK inhibitor, attenuated the beneficial effects of AFW4 on the skin, suggesting that the effects of AFW4 are mediated via p44/42 MAPK activation. Finally, in clinical studies, AFW4 treatment resulted in increased skin hydration and reduced trans-epidermal water loss compared with a placebo group. Collectively, these data provide evidence that AFW4 could be used as a potential therapeutic agent to improve skin barrier damage induced by external stresses.

## 1. Introduction

Skin is the primary defense organ against a harmful external environment. In addition, skin functions as a sensory receptor and regulator of body temperature and moisture content [1,2]. These additional functions of skin contribute to skin homeostasis. The disruption of skin homeostasis leads to skin aging, an emerging global health challenge expected to increase with increasing growth in the aged population. Skin aging, mainly attributed to abnormal cellular physiology, results from extrinsic and intrinsic factors [3]. Intrinsic factors include cellular senescence of skin cells, and extrinsic factors include ultraviolet (UV) irradiation, particulate matter, and chemical and mechanical stress [4,5]. Reportedly, cell proliferation and the moisture retention capability of the skin are related to skin aging because these are closely involved in healthy skin physiology [4] and have been demonstrated to be affected negatively by skin aging [6]. Therefore, maintaining cell proliferation potential and moisture content in the skin is critical to keep skin healthy.

The key molecule involved in skin hydration is hyaluronic acid (HA) [7]. HA production is regulated by the expression of the hyaluronic acid synthase (HAS) genes [8]. Several molecules have been reported to be involved in the expression of HAS genes [9]; for example, retinoic acid (vitamin A) promotes HA production in the epidermis [10], and the increased epidermal HA level contributes to cell proliferation and differentiation in the wound healing process [11]. Furthermore, the natural moisturizing factors (NMFs) are critical regulators to maintain the skin moisture barrier [12]. NMFs include amino acids, HA, filaggrin (FLG), and other molecules affecting skin hydration directly or indirectly [13,14]. Therefore, although several studies focusing on the biosynthesis of NMFs in the skin have been reported, its regulatory mechanisms have not been explored fully.

Vegetable peptones including wheat peptone have been reported to inhibit several enzymes such as angiotensin-converting enzymes, renin, and calmodulin-dependent phosphodiesterase 1 (CaMPDE) [15]. Additionally, they have been used in cell culture medium formulation [16,17]. In addition, in a previous study, our group demonstrated that they promoted the proliferation of adult stem cells and human dermal fibroblasts and increased human type I procollagen synthesis [18]. However, there are no reports on their effects on epidermal cells. In particular, the effects of wheat peptone on keratinocytes have not been examined.

Fermentation is well known to increase the efficacy of the final products by maximizing the concentration of specific active ingredients and has been applied to various industrial fields [19]. For example, Nuruk, a traditional Korean fermentation starter used to produce starch-based alcoholic beverages (takju, cheongju, and soju) using various cereals as raw material, has been reported to improve the production of various active substances in the fermentation process [20]. Nuruk contains several types of microbes, such as bacteria, yeast, and mold [21], that secrete several enzymes, including amylases and proteases, the activity of which results in the production of various active substances in the fermentation process [22]. Among the various microorganisms, we identified Aspergillus oryzae as the main strain in Nuruk and hypothesized that the *A. oryzae*-fermented vegetable peptones could be useful for identifying materials with potential beneficial effects on epidermal cells.

Therefore, in this study, we investigated the effects and mechanisms of action of *A. oryzae*-fermented wheat peptone (AFWP) on the proliferation and hydration of human skin keratinocytes. In addition, a clinical study was performed to ascertain its effects on skin hydration and trans-epidermal water loss (TEWL).

## 2. Results

### 2.1. AFWP Exerted Increased Antioxidant and Proliferation Activities

**Figure 1:**
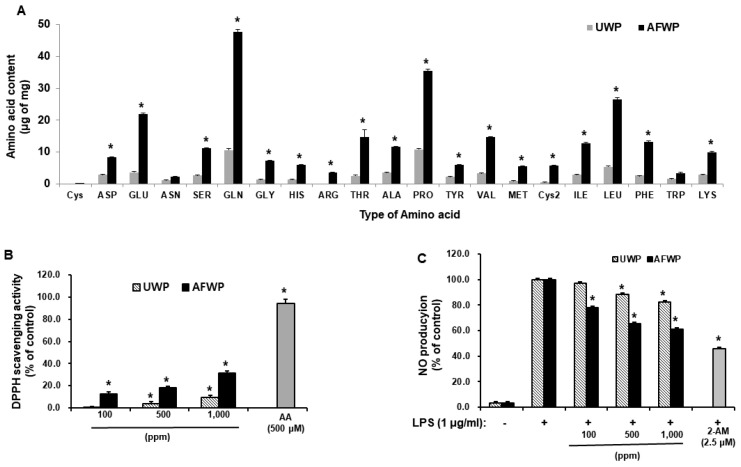
Effects of Aspergillus oryzae-fermented wheat peptone (AFWP) on amino acid composition and antioxidant activity. Effect of AFWP on amino acid composition (**A**), 2,2-diphenyl-1-picrylhydrazyl (DPPH) radical scavenging activity (**B**) and NO production (**C**). (**B**) AFWP or unfermented wheat peptone (UWP) was reacted with DPPH in the dark at 37 °C for 30 min. Absorbance at 517 nm was measured by spectrophotometry. Ascorbic acid (AA) was used as a control compound. (**C**) RAW 264.7 cells were incubated with AFWP or UWP in the presence of lipopolysaccharide (LPS, 1 μg/mL) for 48 h. After the incubation, levels of NO were measured, and 2-amino-4-methylpyridine (2-AM) was used as a control compound. The results were confirmed using four independent experiments. Each experiment was conducted in duplicate. All data are presented as the mean ± SEM of four independent experiments. Statistical significance of differences among the groups was assessed by one-way analysis of variance (ANOVA), followed by Tukey’s multiple-comparison test, using the GraphPad Prism 5 software. * *p* < 0.05 vs. control group. AFWP: Aspergillus oryzae-fermented wheat peptone, UWP: unfermented wheat peptone.

**Figure 2:**
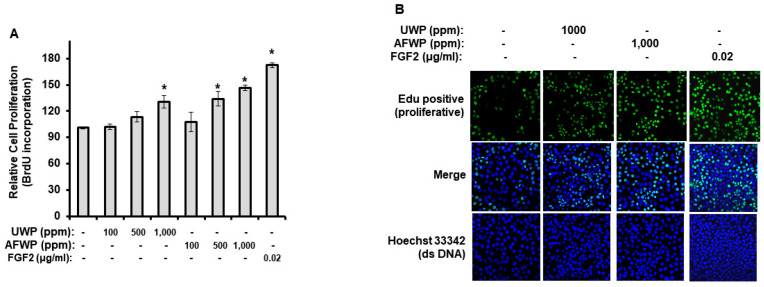
Effects of AFWP on cell proliferation potential. HaCaT cells were incubated with AFWP for 48 h. After incubation, cell proliferation was measured using BrdU cell proliferation assay (**A**) and Edu imaging analysis (**B**). Fibroblast growth factor 2 (FGF2) was used as a control compound. The results were confirmed using four independent experiments. Each experiment was conducted in duplicate. All data are presented as the mean ± SEM of four independent experiments. Statistical significance of differences among the groups was assessed by ANOVA, followed by Tukey’s multiple-comparison test, using the GraphPad Prism 5 software. * *p* < 0.05 vs. control group. AFWP: Aspergillus oryzae-fermented wheat peptone, UWP: unfermented wheat peptone.

As shown in Figure 1A, the amino acid content of AFWP was significantly increased compared with that of UWP (*p* < 0.05). Furthermore, the contents of four amino acids, including glutamic acid, glutamine, proline, and leucine, were remarkably enhanced in AFWP. Next, we investigated the antioxidant activity of AFWP and UWP using DPPH and NO production assays. The antioxidant activity of AFWP was significantly higher than that of UWP. Specifically, AFWP exerted a slightly higher DPPH radical scavenging activity than UWP (Figure 1B), whereas NO production was significantly reduced in AFWP compared to that in UWP (Figure 1C). In addition, as shown in Figure 2, AFWP enhanced the proliferation potential of HaCaT cells, evidenced by BrdU cell proliferation assay (Figure 2A) and EdU staining assay (Figure 2B). Collectively, these data indicate that the fermentation by A. oryzae contributed to the increased antioxidant and cell proliferation activities of wheat peptone.

### 2.2. AFW4 Is the Main Component of AFWP Contributing to Cell Proliferation Potential

**Figure 3:**
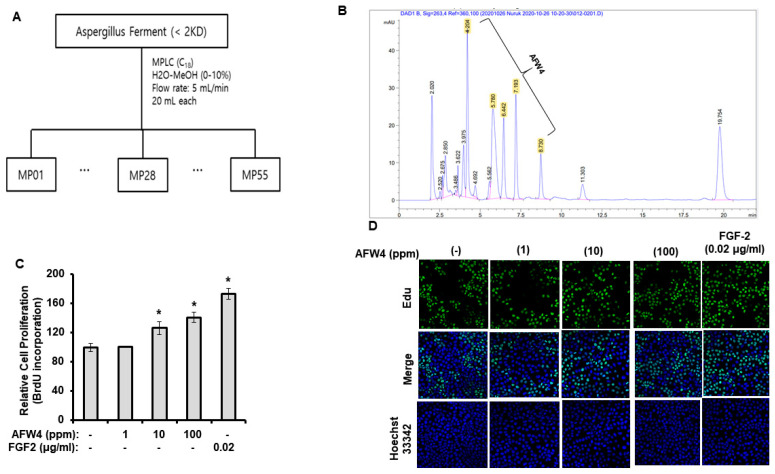
A Wheat peptone fraction of 2 kDa or less (AFW4) promotes cell proliferation potential. (**A**) A scheme to isolate a wheat peptone fraction of 2 kDa or less (AFW4) from AFWP using MPLC. (**B**) HPLC analysis of a wheat peptone fraction of 2 kDa or less. (C and D) HaCaT cells were incubated with AFWP for 48 h. After incubation, cell proliferation was measured using BrdU cell proliferation assay (**C**) and EdU imaging analysis (**D**). Fibroblast growth factor 2 (FGF2) was used as a control compound. The results were confirmed using four independent experiments. Each experiment was conducted in duplicate. All data are presented as the mean ± SEM of four independent experiments. Statistical significance of differences among the groups was assessed by ANOVA, followed by Tukey’s multiple-comparison test, using the GraphPad Prism 5 software. * *p* < 0.05 vs. control group.

To obtain an effective fraction of AFWP, it was separated according to size and polarity using GPC and MPLC, respectively. A fraction of ≤2 kDa was obtained in this process, which was further divided into 55 fractions and grouped into 11 groups (Figure 3A). As shown in Figure 3B, among the grouped fractions, Fr.4 demonstrated the highest cell proliferation potential and was finally chosen for further analysis. Estimation of the cell proliferation potential of Fr. 4 (AFW4) using BrdU cell proliferation assay (Figure 3C) and EdU staining assay (Figure 3D) revealed that AFW4 enhanced the proliferation potential of HaCaT cells in a concentration-dependent manner. These data indicate that AFW4 is the main component contributing to the cell proliferation potential of AFWP. Furthermore, we performed peptide sequencing to examine amino acid sequences of AFW4. The peptide sequencing for AFW4 identified six kinds of low molecular weight peptides, each consisting of 4–10 amino acids (Table 1).

### 2.3. AFW4 Contributes to Skin Hydration through the Upregulation of NMF Levels

**Figure 4:**
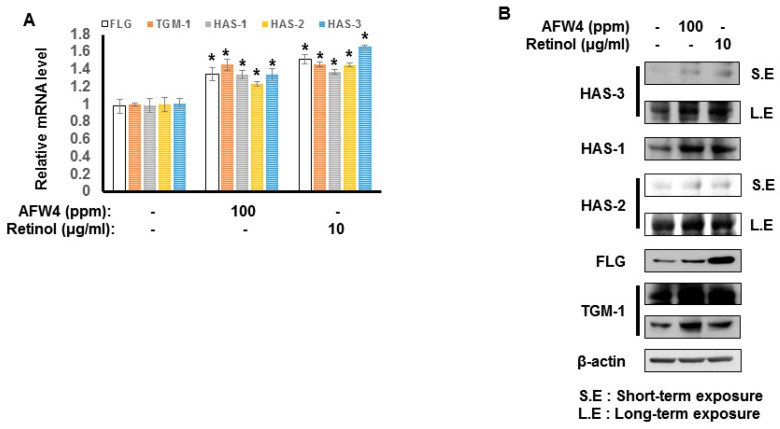
Effect of AFW4 on the expression of NMF-related genes. HaCaT cells were incubated with AFW4 for 48 h. Afterward, the cells were harvested and subjected to real-time RT-PCR analysis (**A**) and Western blotting (**B**) to analyze mRNA and protein expression levels of NMF-related genes, respectively. Retinol was used as a control compound. The results were confirmed using four independent experiments. Each experiment was conducted in duplicate. All data are presented as the mean ± SEM of four independent experiments. Statistical significance of differences among the groups was assessed by ANOVA, followed by Tukey’s multiple-comparison test, using the GraphPad Prism 5 software. * *p* < 0.05 vs. control group.

**Figure 5:**
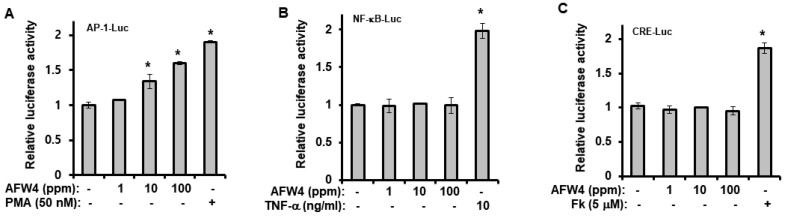

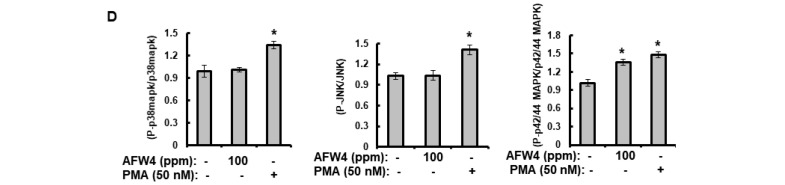
Effects of AFW4 on cell proliferation and skin hydration are mediated by activating p44/42 MAPK. (**A**–**C**) HaCaT cells were co-transfected with the AP-1-, NF-κB-, or CRE-promoter-luciferase reporters and β-galactosidase reporter vector using polyethylenimine. After 24 h, the transfected cells were incubated with AFW4, phorbol 12-myristate 13-acetate (PMA), tumor necrosis factor (TNF)-α or forskolin (Fk). Twenty-four hours after the incubation, the cells were harvested and subjected to luciferase reporter assay. (**D**) Cells were incubated with AFW4 or PMA for 1 h. The cells were harvested immediately after the incubation, and the protein levels of MAPKs and their phosphorylated forms were detected by ELISA. The results were confirmed using four independent experiments. Each experiment was conducted in duplicate. All data are presented as the mean ± SEM of four independent experiments. Statistical significance of differences among the groups was assessed by ANOVA, followed by Tukey’s multiple-comparison test, using the GraphPad Prism 5 software. * *p* < 0.05 vs. control group.

We examined the effects of AFW4 on the biosynthesis of NMFs in HaCaT cells. AFW4 increased mRNA and protein levels of NMF-related genes, including FLG, TGM-1, HAS-1, HAS-2, and HAS-3 (Figure 4A,B), indicating that AFW4 contributes to the biosynthesis of NMFs in the skin. Furthermore, to elucidate the mechanisms underlying the AFW4-induced upregulation of NMF-related genes, AP-1-, NF-κB-, and CRE-promoter-luciferase and β-galactosidase assays were performed. The results showed that AFW4 did not affect NF-κB and CRE reporter activities, whereas AP-1 reporter was activated by AFW4 (Figure 5A–C). Phorbol 12-myristate 13-acetate (PMA) and tumor necrosis factor (TNF)-α were used as the positive controls in the AP-1 and NF-κB reporter assays, respectively. Forskolin was used to stimulate adenylate cyclase in the CRE reporter assay. In addition, among MAPKs, AFW4 increased the phosphorylation levels of p44/42 MAPK. However, phosphorylation levels of p38 MAPK and JNK were not affected by AFW4 (Figure 5D). Collectively, these data suggest that the AFW4-induced expression of NMF-related genes could be mediated via the activation of p44/42 MAPK.

### 2.4. AFW4 Induces Cell Proliferation and Expression of NMF-Related Genes through p44/42 MAPK Activation

**Figure 6:**
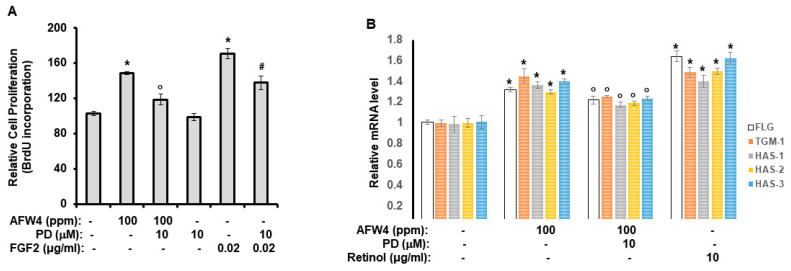
PD98059 attenuates AFW4 effects on the cell proliferation and expression of NMF-related genes. (**A**) HaCaT cells were incubated with AFW4 in the presence of PD98059 (PD) for 48 h. After the incubation, cell proliferation was measured using BrdU cell proliferation assay. Fibroblast growth factor 2 (FGF2) was used as a control compound. (**B**) HaCaT cells were incubated with AFW4 in the presence of PD for 48 h. After the period, the cells were harvested and subjected to real-time RT-PCR analysis. Retinol was used as a control compound. All data are presented as the mean ± SEM of four independent experiments. The results were confirmed using four independent experiments. Each experiment was conducted in duplicate. Statistical significance of differences among the groups was assessed by ANOVA, followed by Tukey’s multiple-comparison test, using the GraphPad Prism 5 software. * *p* < 0.05 vs. control group, ° *p* < 0.05 vs. AFW4-treated group, ^#^ *p* < 0.05 vs. FGF2-treated group.

To confirm the involvement of p44/42 MAPK in the cell proliferation and expression of NMFs in HaCaT cells, we incubated the HaCaT cells with AFW4 in the presence of PD98059, a p44/42 MAPK inhibitor. As shown in Figure 6A, AFW4 increased the cell proliferation potential, which was attenuated by PD98059. Similarly, PD98059 reduced the proliferation potential of FGF2. In addition, the AFW4-induced upregulation of NMF-related genes was reduced by PD98059 treatment (Figure 6B). These results provide evidence that the effects of AFW4 on cell proliferation and NMF biosynthesis are mediated by p44/42 MAPK activation.

### 2.5. AFW4 Exerts Antioxidant Activity

**Figure 7:**
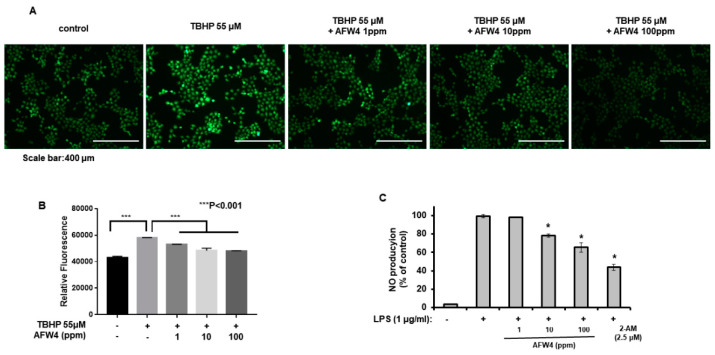
AFW4 exerts antioxidant activity in HaCaT cells. (**A** and **B**) HaCaT cells were incubated with AFW4 in the presence of TPHP (55 μM) for 24 h and then subjected to fluorescence image analysis (**A**). In addition, densitometric analysis (**B**) for fluorescence images was conducted. All data are presented as the mean ± SEM of four independent experiments. (**C**) RAW 264.7 cells were incubated with AFW4 in the presence of lipopolysaccharide (LPS, 1 μg/mL) for 48 h. After incubation, the levels of NO were measured, and 2-amino-4-methylpyridine (2-AM) was used as a control compound. The results were confirmed using four independent experiments. Each experiment was conducted in duplicate. All data are presented as the mean ± SEM of four independent experiments. Statistical significance of differences among the groups was assessed by ANOVA, followed by Tukey’s multiple-comparison test, using the GraphPad Prism 5 software. * *p* < 0.05 vs. LPS-treated group.

As described earlier, AFWP showed a stronger antioxidant activity than UWP. Therefore, to examine whether AFW4 was the key component contributing to the strong antioxidant activity of AFWP, we estimated the effects of AFW4 on the production of ROS and NO. As shown in Figure 7A, TBHP-induced production of ROS was reduced upon AFW4 treatment in a concentration-dependent manner. In addition, LPS-induced production of NO was significantly reduced (Figure 7B), suggesting that AFW4 is the key factor responsible for the antioxidant activity of AFWP.

### 2.6. AFW4 Improves Skin Hydration and TEWL

#### 2.6.1. Moisture Content

Both AFWP4 and AFWP CON significantly improved skin moisturization 2 and 4 weeks after use of the test products compared to that before use (Table 2, AFWP4: *p* < 0.01, AFWP CON: *p* < 0.001), wherein AFWP4 revealed a significantly higher rate of improvement both after 2 and 4 weeks of application than AFWP CON (Table 3; *p* < 0.01 and *p* < 0.05, respectively). In addition, neither AFWP4 nor AFWP CON induced side effects such as redness, itching, irritation, or any other signs.

#### 2.6.2. Trans-Epidermal Water Loss (TEWL)

For AFWP4, the TEWL decreased significantly on 2 and 4 weeks after use compared to that before use (*p* < 0.05 and *p* < 0.01, respectively), and for AFWP CON, the reduction was significant only after 4 weeks (*p* < 0.05) of use compared to that before use (Table 4). Additionally, the rate of TEWL improvement was higher both 2 and 4 weeks after AFWP4 application compared to that with AFWP CON application; however, it was statistically significant only after week 4 (Table 5; *p* < 0.05). Collectively, these data indicate that AFWP4 increases skin hydration and reduces TEWL, leading to moisturization in the skin.

## 3. Discussion

**Figure 8:**
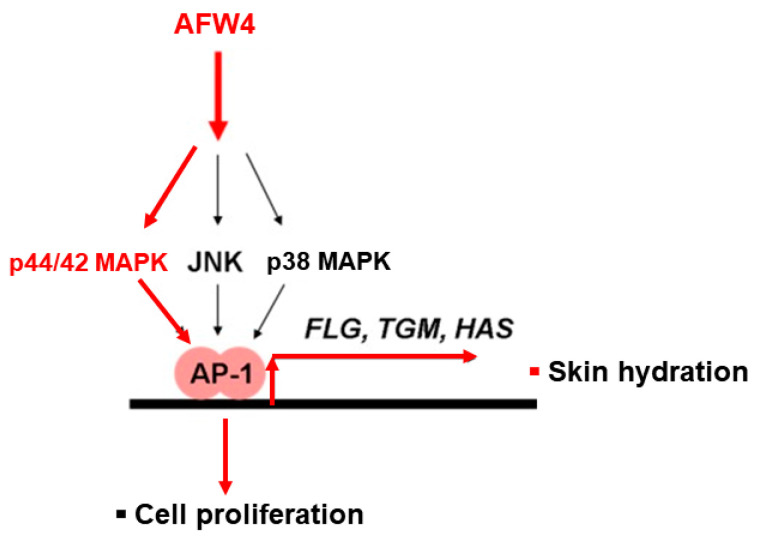
Mechanisms of cell-proliferating and skin-hydrating activities of AFW4. AFW4 promoted cell proliferation and upregulated expression of skin hydrating genes in HaCaT cells. The effects of AFW4 on cell proliferation and skin hydration are mediated by activating p44/42 MAPK. Red arrow: Activation pathway by AFW4.

This study demonstrates the antagonizing effects of AFWP on skin cell phenotypes such as reduced cell proliferation and moisture content, the hallmarks of skin aging. Particularly, AFW4, a specific low molecular weight wheat peptone fraction (<2 kDa) comprising six peptides, each 4–6 amino acids long, showed better efficacy in the cell proliferation potential. AFW4 promoted the antioxidant and proliferation potential of human keratinocytes, as evidenced by the BrdU cell proliferation assay in HaCaT cells. In addition, AFW4 treatment upregulated the expression of NMF-related genes, including FLG, TGM-1, and HAS-1–3, as demonstrated by real-time RT-PCR and Western blotting analyses. Furthermore, the findings unraveled the underlying mechanism of the promoting effects of AFW4 on cell proliferation and skin hydration and demonstrated that the promoting effects of AFW4 are mediated via the activation of p44/42 MAPK, as summarized in Figure 8.

Water is essential for the normal physiology of the skin and particularly its outermost layer, the stratum corneum (SC) [23]. Therefore, water loss from the skin is elaborately regulated, and this function is dependent on the complex nature of the SC. It has been reported that two major components critically contribute to the moisture content in the SC [24]. The first component comprises the SC intercellular lipids, which are orderly arranged to form a skin barrier to TEWL, whereas the natural hygroscopic agents form the second component within the keratinocytes in the SC (corneocytes), collectively referred to as NMFs. The maintenance of an appropriate moisture content in the SC contributes to SC maturation and skin desquamation [25]. On the contrary, abnormally increased TEWL impairs various enzymatic activities required for normal barrier formation and desquamation, leading to accelerated skin aging and induction of skin diseases [26]. Therefore, maintenance of proper water content in the keratinocyte is essential for normal skin homeostasis. Several studies on the mechanisms of skin hydration and related materials have recently been conducted. In this study, we demonstrated that AFW4 induced the upregulation of NMF-related genes such as FLG, TGM-1, and HAS-1–3 in keratinocytes. In addition, the clinical study showed that AFW4 improved skin hydration and decreased TEWL involved in skin barrier function. Taken together, these results provide evidence that AFW4 could potentially contribute to the maintenance of normal SC structure and epidermal barrier function.

Cell proliferation or cellular senescence has been considered as one of the main hallmarks of skin aging [27]. Recent studies have reported the involvement of senescent cells in skin aging and aging-related diseases [28,29]. Furthermore, cell proliferation and differentiation in the skin need to be well controlled to maintain healthy skin. However, various stresses disrupt the balance between proliferation and differentiation, leading to skin aging and skin diseases [30,31]. In addition, cell proliferation potential is weak in the aged skin. Therefore, therapeutic approaches for skin aging include the clearance of senescent cells via the use of senolytics or an increase in the cell proliferation potential of skin cells. A previous study reported that vegetable peptones can induce cell proliferation and that the mechanisms underlying these actions may be mediated via the Raf- p44/42 MAPK-p90 ribosomal s6 kinase- CCAAT/enhancer binding protein β activation pathway [32]. In this study, we demonstrated that AFW4 significantly promotes cell proliferation potential, and its effect is mediated by the activation of p44/42 MAPK. Although the mechanism of action of AFW4 is not the same as that of vegetable peptones, it can induce cell proliferation, suggesting the potential for its use in therapeutics developed to improve skin aging.

Finally, we demonstrated that AFW4 activated AP-1 signaling, but not NF-κB- and CRE-dependent signaling. In the AP-1 signaling, while AFW4 had no effects on p38 MAPK and JNK activation, it induced the phosphorylation of p44/42 MAPK. In addition, the promoting effects of AFW4 on cell proliferation and expression of NMF-related genes were attenuated by PD98059, a p44/42 MAPK inhibitor. Collectively, these data provide evidence that AFW4 contributes to cell proliferation and skin hydration by activating p44/42 MAPK.

## 4. Materials and Methods

### 4.1. Materials

Dulbecco’s modified Eagle’s medium (DMEM), fetal bovine serum (FBS), phosphate-buffered saline (PBS), and penicillin-streptomycin were purchased from HyClone (Logan, UT, USA). Ascorbic acid, 1-diphenyl-2-picryl-hydrazyl (DPPH), and TRIzol were purchased from Sigma Aldrich Chemical Co., (St. Louis, MO, USA). Antibodies against phospho- or total forms of p38 mitogen-activated protein kinases (MAPK), p44/42 MAPK, c-Jun N-terminal kinase (JNK), and β-actin were obtained from Cell Signaling Technology (Beverly, MA, USA). Anti-HAS-2 antibody was purchased from Santa Cruz Biotechnology (Santa Cruz, CA, USA).

### 4.2. Sample Preparation

#### 4.2.1. Preparation of AFWP

Anjeunbaengi wheat (*Triticum aestivum* L.), also known as Jinju native wheat, was purchased from Jinju Gokja Institute Co., (Korea) and ground into flour. The powder was kneaded with the half volume of distilled water (DW). Aspergillus oryzae, cultured in potato dextrose broth (100 mL), was inoculated to the paste and incubated at 37 °C for 24 h. Afterward, the fermented dough was cut into small slices, the same volume of DW was added, and the dough was re-incubated at 37 °C for 24 h. After incubation, 3X volume of DW was added to the fermented dough, and the slurry formed was filtered using 0.45 μm filter membranes (Hyundai Micro Co., Korea). The filtered liquid was concentrated by an evaporator at 60 °C and 40 bars to obtain a 10 Brix solution, and the concentrated solution was used as the fermented product of *A. oryzae*, referred hereafter as to AFWP.

#### 4.2.2. Preparation of Low Molecular Weight Wheat Peptone

The AFWP was mixed with 3 volumes of ethanol (100%) and incubated at 4 °C overnight. After the incubation, it was concentrated to 10 Brix to remove ethanol. Subsequently, ultrafiltration was performed using a 2 kDa Ultra Filter (Sartorius Stemdim Lab Co., Göttingen, Germany) at 3000× *g* for 70 min to obtain the low molecular weight samples (<2 kDa). The filtered fluid was purified by precipitation. In order to remove impurities other than protein, to this solution, 1 N NaOH was added to adjust the pH to 6, and citric acid was added to adjust the pH to 3.99. Subsequently, the purified supernatant was filtered using a 0.45 μm nylon filter, and the filtrate was lyophilized to a powder form.

### 4.3. Gel Permeation Chromatography (GPC) Analysis

GPC analysis was performed using an EcoSEC HLC-8320 GPC system fitted with a refractive index detector (Tosoh Bioscience LLC, King of Prussia, PA, USA). The separation was achieved using 0.1 M NaNO_3_ (mobile phase) with a flow rate of 1 mL/min and TSKgel GMPWxl and TSKgel G2500PWxl (7.8 × 300 mm) columns at 40 °C. The sample obtained in Section 4.2.1 or Section 4.2.2 was reconstituted in 100 mL and filtered using a 0.45 μm nylon filter. The filtrate (100 μL; 3 mg/mL) was then injected. The standard material used was polyethylene glycol (PEG)/polyethylene oxide (PEO) or polysaccharide. The result was analyzed by EcoSEC software (Tosoh Bioscience LLC, King of Prussia, PA, USA).

### 4.4. Amino Acid Composition Analysis

The unfermented wheat peptone (UWP) and AFWP were freeze-dried to a powder form and dissolved in 1 mL water. Thereafter, 30 μL of each solution was dried and derivatized with phenylisothiocyanate (PITC) (MeOH: H_2_O: tetraethyl ammonium (TEA): PITC = 7:1:1:1) at room temperature for 30 min. After drying the reactant, it was melted using 140 mM sodium acetate trihydrate, 0.15% TEA, 0.03% ethylenediaminetetraacetic acid (EDTA), and 6% CH3CN, at pH 6.1, followed by centrifugation (4 °C, 10 min, 4000 rpm). The supernatant was then filtered using a 0.45 μm nylon filter (Acrodisc, Pall Co., New York, NY, USA) and used for HPLC analysis. The HPLC analysis was performed on an Agilent 1260 Series HPLC system using Waters Nova-Pak C18, column (4 μm, 3.9 × 300 mm) and HP 1100 Series (Agilent), 254 nm detector. The mobile phase comprised solution A (140 mM sodium acetate trihydrate, 0.15% TEA, 0.03% EDTA, 6% CH3CN, pH 6.1) and solution B (60% CH3CN, 0.015% EDTA).

### 4.5. Medium-Pressure Liquid Chromatography (MPLC) Analysis

AFWP was separated according to polarity through medium pressure liquid chromatography (MPLC) using a Sepacore Chromatography System (BUCHI Co., Flaville, Switzerland) on a C18 silica gel based column with a cartridge size 80 g. For MPLC, 20 mL of the sample was eluted under gradient conditions using a H2O-MeOH (10%–100%) solvent [33].

### 4.6. Peptide-Sequencing Analysis

In order to examine amino acid sequence of AFW4, the peptide-sequencing analysis was conducted at the Life Science Laboratory Co., (EMASS, Korea) using a Thermo Fisher Dionex UHPLC Ultimate 3000 system coupled to a mass spectrometer with an ESI source and an ACQUITY UPLC column (C18, 1.7 μm). Purification of compounds was performed with an AB Sciex Q-TOF system. The analysis was performed with a linear gradient of solvents, at a flow rate of 300 μL/min, as follows: 0–95 min, from 99% H_2_O (0.1% formic acid): 1% ACN (0.1% formic acid) to 50% H_2_O (0.1% formic acid): 50% ACN (0.1% formic acid); 95–105 min, from 50% H_2_O (0.1% formic acid): 50% ACN (0.1% formic acid) to 100% ACN (0.1% formic acid). The detected peptides were searched in the Uniprot database for substrate proteins in T. aestivum, and the possible sequences of the peptides were obtained [34].

### 4.7. 2,2′-Diphenyl-1-picrylhydrazyl (DPPH) Radical Scavenging Activity

The ability of the extracts to annihilate the DPPH radical was investigated by the method described by Blois (1958) [35]. An equal volume of the sample (100, 500, and 1000 ppm of AFWP or 500 μM ascorbic acid) was added to a methanolic solution of DPPH (0.2 mM) in 96-well plates and incubated for 30 min at room temperature. Afterward, the absorbance was recorded at 517 nm. The experiment was repeated four times. Ascorbic acid (500 μM) was used as the standard control.

### 4.8. Cell Culture

HaCaT cells and RAW 264.7 cells were obtained from Cell Lines Service (CLS, Eppelheim, Germany). The HaCaT cells and RAW 264.7 cells were cultured in Dulbecco’s Modified Eagle’s Medium (DMEM) high glucose with 10% FBS and 1% penicillin-streptomycin at 37 °C in a humidified atmosphere containing 5% CO_2_. The cells were sub-cultured every 2–3 days using 0.5% trypsin-EDTA solution.

### 4.9. Cell Proliferation

HaCaT cells were seeded at 5 × 10^3^ cells/well in 96-well plates in the complete medium. After 24 h of incubation, cells were incubated with the indicated concentrations of the test materials (100, 500, and 1000 ppm of AFWP and UWP or 1, 10, and 100 ppm of AFW4) for 2 days under serum-free conditions (in DMEM devoid of serum, at 37 °C with 5% CO_2_). The serum-free conditions were chosen to exclude the unknown effects of the exogenous serum, the compositions of which might vary depending on the donor species, the age and feedstock of the animal the serum was obtained from, and the season. After 2 days, cell proliferation was measured using the bromodeoxyuridine (BrdU) incorporation assay. The ELISA-based detection of the BrdU incorporation was performed using the BrdU Cell Proliferation Assay Kit (Cell Signaling Technology, Danvers, MA, USA) according to the manufacturer’s instructions.

### 4.10. Quantification of Nitric Oxide Levels

Nitric oxide production was estimated by measuring the nitrite levels in the supernatants of cultured RAW 264.7 cells. The cells were seeded at a density of 1.5 × 10^5^ cells/mL in 48-well plates and cultured for 24 h. The cells were stimulated with lipopolysaccharide (LPS) (1 μg/mL) and treated with the test samples (100, 500, and 1000 ppm of AFWP and UWP or 1, 10, and 100 ppm of AFW4) for 24 h. Afterward, the supernatant was mixed with an equal volume of Griess reagent (Sigma Aldrich Chemical Co., St. Louis, MO, USA.) and incubated at room temperature for 10 min. The nitrite concentrations were determined at an optical density of 540 nm using a microplate reader (Molecular Devices, San Jose, CA, USA).

### 4.11. Western Blotting Analysis

HaCaT cells were seeded onto 60 mm cell plates. After 24 h, the cells were incubated with AFW4 (100 ppm) or retinol (10 μg/mL) for 48 h. The cells were harvested and centrifuged for 5 min at 13,000 rpm. The supernatant was discarded, and the cells were lysed with RIPA lysis buffer [25 mM Tris-HCl (pH 7.6), 150 mM NaCl, 1% NP-40, 1% sodium deoxycholate, 0.1% SDS (Thermo Fisher Scientific, Waltham, MA, USA)] containing Halt protease and a phosphatase inhibitor cocktail (Thermo Fisher Scientific). The proteins extracted from the cells were separated by 8%–10% SDS electrophoresis and transferred onto nylon membranes. The membranes were blocked with 5% skim milk for 1 h and then incubated with primary antibodies (filaggrin, transglutaminase-1, hyaluronic acid synthase 1–3, or β-actin) at 4 °C overnight. The membranes were washed thrice with Tris-buffered saline (TBS) containing Tween 20 and probed with secondary antibodies for 1 h at room temperature. The blots were visualized using ECL Western Blotting Detection Reagents (Biorad, Hercules, CA, USA).

### 4.12. MAPK-Phosphorylation Analysis

HaCaT cells were incubated with AFW4 (100 ppm) or PMA (50 nM) for 1 h. The levels of phospho-SAPK/JNK (Thr183/Tyr185), phospho-p38 MAPK (Thr180/Tyr182), JNK, and p38 MAPK were measured using the PathScan Inflammation Multi-Target Sandwich ELISA Kit (Cell Signaling Technology) according to the manufacturer’s instructions. The levels of the phospho-p42/44 MAPK (Thr202/Tyr204) and p42/44 MAPK expressions were also determined using the PathScan Cell Growth Multi-Target Sandwich ELISA Kit (Cell Signaling Technology, Danvers, MA, USA) according to the manufacturer’s instructions.

### 4.13. Analysis of mRNA Levels Using Real-Time Quantitative Reverse Transcription-Polymerase Chain Reaction (RT-PCR)

HaCaT cells were incubated with AFW4 (100 ppm) or retinol (10 μg/mL) for 48 h. Afterward, the cells were harvested and subjected to real-time RT-PCR analysis. RT-PCR analysis was conducted as previously described [36] using an ABI7900HT Instrument (Applied Biosystems, Waltham, MA, USA). For TaqMan analysis, predesigned or optimized assays on demand (Applied Biosystems) were used, including FLG (ID: Hs06628971_s1), transglutaminase-1 (TGM-1) (ID: Hs00165929_m1), HAS-1 (ID: Hs00377726_m1), HAS-2 (ID: Hs01552331_m1), HAS-3 (ID: Hs04187819_g1), glyceraldehyde-3-phosphate dehydrogenase (GAPDH) (ID: Hs00266705_g1), hypoxanthine-guanine phosphoribosyltransferase (HPRT) (Hs02800695_m1), and 18S (Hs03003631_g1). The data were analyzed using ABI Sequence Detector Software version 2.0 (Applied Biosystems). Total RNA was extracted from cells using TRI reagent^®^ (Sigma Aldrich Chemical Co.) according to the manufacturer’s instructions and stored at −70 °C until use. cDNA was synthesized from total RNA (1 μg) using Moloney murine leukemia virus (MuLV) reverse transcriptase (ThermoFisher scientific, Waltham, MA, USA) according to the manufacturer’s instructions. Real-time RT-PCR analysis was conducted as previously described [37]. The results were normalized to the expression level of endogenous GAPDH and two additional housekeeping genes (18S and HPRT). Expression levels of target genes were normalized to those observed in controls. Results were verified through four-time repetition of the same experiment, each of which was conducted in triplicate.

### 4.14. Luciferase Reporter Assay

Luciferase reporter assay and β-galactosidase assay were performed to determine the promoter activities at the transcriptional level [38]. NF-κB (Stratagene, La Jolla, CA, USA), CRE (Stratagene), and AP-1 (Stratagene) promoter-firefly luciferase reporters were used. Cells were seeded onto 60 mm cell plates and incubated at 37 °C overnight. The cells were co-transfected with 1.5 μg of the promoter-luciferase reporters and 1.5 μg of the β-galactosidase vector (Promega Corporation) using 7.5 μg polyethylenimine (Sigma-Aldrich, St. Louis, MO, USA) to quantify the promoter–luciferase activities. Four hours after transfection, the cells were cultured in fresh medium for 24 h and then incubated with AFW4 (1, 10, and 100 ppm), phorbol 12-myristate 13-acetate (PMA, 50 nM), tumor necrosis factor (TNF)-α (10 ng/mL) or forskolin (Fk, 5 μM). Twenty-four hours after incubation, the cells were harvested and subjected to a luciferase reporter assay. The β-galactosidase activity was assayed using the β-galactosidase enzyme assay system (Promega Corporation). Briefly, after 24 h of incubation at 37 °C, the cells were harvested with PBS and lysed with the reporter lysis buffer (Promega Corporation). The cells were then centrifuged, and the supernatants were transferred into 96-well plates. Subsequently, 1 M sodium carbonate was added to the wells to stop the color development, and the absorbance of the samples was measured at 420 nm using a microplate reader (BioTek, Winusky, VT, USA) to quantify the β-galactosidase activity. Luciferase activity was assayed using the luciferase activity assay system (Promega Corporation, Madison, WI, USA). The cells were harvested with PBS and lysed with the reporter lysis buffer (Promega Corporation). The cells were then centrifuged, and the supernatants were transferred into 96-well plates. Luciferase assay substrate and luciferase assay buffer were added to the wells, and the luminescence was determined using a microplate reader (BioTek, Winooski, VT, USA). Luciferase activity was expressed as the ratio of promoter-dependent firefly luciferase activity to β-galactosidase activity.

### 4.15. Cellular Reactive Oxygen Species (ROS) Detection Assay

ROS production was quantitatively measured using the 2ʹ,7ʹ-Dichlorofluorescin Diacetate (DCFDA)-cellular ROS detection assay kit (ab113851) and analyzed using fluorescence microscopy and a microplate reader (BioTek, Winooski, VT, USA). HaCaT cells were seeded into 60-mm cell plates or 96-well plates. The cultured cells were treated with AFW4 (1, 10, and 100 ppm) in the presence of tert-butyl hydroperoxide (TBHP, 55 μM) solution, which was used as a positive control. After 24 h, they were washed twice in PBS and stained with 25 µM DCFDA in PBS for 15 min at 37 °C in the dark. The stained cells were washed, and their fluorescence signals were detected at an Ex/Em of 485/535 nm. The change in fluorescence was determined as the percentage of the control fluorescence after background subtraction.

### 4.16. Clinical Evaluation and Study Design

To determine the efficacy of *A. oryzae*-fermented wheat, TEWL and hydration efficiency were evaluated by human skin tests using 11 woman volunteers (aged 19–40 years) with dry skin. The experiment was per the regulation of the Ministry of Food and Drug Safety, Korea. Briefly, two test products were prepared without AFW4 (AFWP CON as placebo) and with 1.0% AFW4 in an aqueous solution containing 6.0% butylene glycol, 3.0% pentylene glycol, and 0.1% ethylhexylglycerin. The test products were randomly applied to the cheeks (below the cheekbone between the nose and ear) of all 11 women twice a day for 4 weeks. Specifically, the control product was applied to the right cheek of the volunteers, and the AFWP4 product was applied to the left. A subjective questionnaire and skin tests were conducted before and after 2 and 4 weeks of applying the test products. The study was approved (IRB certification No.: 2020021201-202103-HR-001-01, dated: 8 March 2021) by the Institutional Review Board Committee of CRA Korea Inc. (Chungbuk, Korea), and written informed consent was obtained from each volunteer.

#### 4.16.1. Skin Hydration

Moisture content was measured using Skin-O-Mat^®^ (COSMOMED, Wetter, Germany) on the cheek area before application and 2 and 4 weeks after application. During measurement, a Corneometer probe was set to be in contact with the skin. The measurement was done three times through the sensor. The average value was used to evaluate the skin moisture content [39].

#### 4.16.2. TEWL

TEWL was measured in the same cheek area using the Vapometer (Delfin, Finland) before and after applying the test product. The amount of water loss on the epidermis, which predicts the water retention capacity of the skin, is an indicator of skin water barrier functions. This indicates the amount of water loss through the skin (g/m^2^ h). TEWL was measured three times for 10 s, and the average value was used for evaluation [18,40].

#### 4.16.3. Statistical Analyses of Clinical Data

The clinical statistical analysis result was confirmed to be significant at the 95% confidence interval. Normality verification for the results was performed using the Kolmogorov–Smirnov and Shapiro–Wilk methods. After normality verification, the comparison before and after the test to meet the normality was analyzed using the paired *t*-test, a parametric test; when the normality was not satisfied, the Wilcoxon signed-rank test, a non-parametric test, was used. In the case of the intergroup comparison, when normality was satisfied, an independent *t*-test, a parametric test, was used, and when normality was not satisfied, a nonparametric test, Mann–Whitney U test, was used. All clinical statistical analyses were performed using SPSS software (IBM SPSS Statistics, Armonk, NY, USA).

### 4.17. Statistical Analyses of Experimental Data

The data are expressed as the mean ± standard error of the mean (SEM). Analyses of differences between two groups were performed using Student’s *t*-test. The comparison between multiple groups was performed using one-way analysis of variance (ANOVA), followed by Tukey’s multiple-comparison test, for which the GraphPad Prism (5.0) (GraphPad, La Jolla, CA, USA.) software was used. Statistical significance was considered when the *p*-value was less than 0.05.

## 5. Conclusions

This study demonstrated that AFW4, a low molecular weight fraction comprising six peptides of 4–6 amino acids, antagonizes against reduced cell proliferation and moisture content in aged skin cells and has slight antioxidative effects. These effects of AFW4 were mediated via the activation of p44/42 MAPK. In addition, the clinical study showed significant improvement in skin hydration and skin barrier function. Our results suggest that AFW4 could be used as a potential therapeutic agent for ameliorating the skin aging symptoms induced by various stresses on the skin.

## Figures and Tables

**Table 1:** The peptide sequences of AFW4 inferred from Uniprot (*Triticum aestivum*).

Sample Name	Database	Enzyme Used for Hydrolysis	The Most Suitable Amino Acid Sequence
AFW4	Uniprot Database: Organism: *T. aestivum* (Wheat)	Random	MASI
			TMIT
		Trypsin-Chymotrypsin (K, R, W, H, M, L, Y/X-X)	MLSLF YGSD LPQHSRDSLR AAQPR

**Table 2:** Effects of AFWP on skin moisture content (*n* = 11).

Sample	Mean ± SEM			*p*-Value	
	0 Week	2 Weeks	4 Weeks	0 vs. 2 Weeks	0 vs. 4 Weeks
AFWP4	23.3 ± 1.32	28.1 ± 1.68	31.8 ± 1.45	0.003 **	0.003 **
AFWP CON	24.1 ± 1.61	26.3 ± 1.68	29.5 ± 1.24	0.000 ^###^	0.000 ^###^

SEM, Standard error of mean; ** *p* < 0.01, obtained by Wilcoxon signed-rank test; ### *p* < 0.001 obtained by paired *t*-test.

**Table 3:** Skin moisture content improvement rate (%).

Weeks	Mean ± SEM		Title 4
	AFWP4	AFWP CON	AFWP4 vs. AFWP CON
2	20.6 ± 2.15	9.7 ± 1.89	0.003 **
4	38.3 ± 4.27	25.7 ± 5.33	0.017 *

SEM, Standard error of mean; * *p* < 0.05, ** *p* < 0.01 obtained by the Mann–Whitney U test.

**Table 4:** Effects of AFWP4 on trans-epidermal moisture loss (TEWL).

Sample	Mean ± SEM			*p*-Value	
	0 Week	2 Weeks	4 Weeks	0 vs. 2 Weeks	0 vs. 4 Weeks
AFWP4	18.8 ± 1.43	17.4 ± 1.30	15.9 ± 1.14	0.010 *	0.003 **
AFWP CON	18.9 ± 1.15	18.3 ± 1.05	17.3 ± 1.07	0.167	0.008 **

SEM, Standard error of mean; * *p* < 0.05, ** *p* < 0.01, obtained by Wilcoxon signed-rank test.

**Table 5:** Trans-epidermal moisture loss (TEWL) improvement rate (%).

Weeks	Mean ± SEM		Title 4
	AFWP4	AFWP CON	AFWP4 vs. AFWP CON
2	7.3 ± 2.08	2.6 ± 1.76	0.103
4	14.8 ± 2.25	8.2 ± 2.04	0.042 *

SEM, Standard error of mean; * *p* < 0.05, obtained by Wilcoxon signed-rank test.

## Data Availability

Not applicable.
